# Bone marrow-derived mesenchymal stem cells protect against cisplatin-induced acute kidney injury in rats by inhibiting cell apoptosis

**DOI:** 10.3892/ijmm.2013.1517

**Published:** 2013-10-08

**Authors:** SHAOHUA QI, DONGCHENG WU

**Affiliations:** Department of Biochemistry and Molecular Biology, School of Basic Medical Sciences, Wuhan University, Wuhan, Hubei 430071, P.R. China

**Keywords:** cisplatin, mesenchymal stem cells, acute kidney injury

## Abstract

Acute kidney injury (AKI) is a common syndrome with a high mortality and morbidity rate. Recent developments in stem cell research have shown great promise for the treatment of AKI. The aim of this study was to investigate the therapeutic potential and anti-apoptotic mechanisms of action of bone marrow-derived mesenchymal stem cells (BM-MSCs) in the treatment of AKI induced by cisplatin *in vivo* and *in vitro. In vivo*, adult male Sprague-Dawley rats (n=24) were administered BM-MSCs intravenously one day after cisplatin injection. The rats were sacrificed four days after the cisplatin injection and the effects of BM-MSCs on cisplatin-induced AKI, as well as the anti-apoptotic mechanisms involved were investigated. *In vitro*, NRK-52E cells, a rat renal proximal tubular cell line, were incubated in conditioned medium or complete medium in the presence or absence of cisplatin, followed by cell proliferation and apoptosis assays. The infusion of BM-MSCs preserved renal function, ameliorated renal tubular lesions, reduced apoptosis and accelerated tubular cell regeneration in the rats with cisplatin-induced AKI. The infusion of BM-MSCs also inhibited the activation of two mitogen-activated protein kinases, p38 and ERK, downregulated the expression of Bax and cleaved caspase-3, and upregulated the expression of Bcl-2. BM-MSC-conditioned medium improved NRK-52E cell viability and inhibited apoptosis. In conclusion, our results demonstrate that injecting rats with BM-MSCs protects renal function and structure in cisplatin-induced AKI by inhibiting cell apoptosis *in vivo*. BM-MSC-conditioned medium protects renal cells from apoptosis induced by cisplatin *in vitro*. Hence, the infusion of BM-MSCs should be considered as a possible therapeutic strategy for the treatment of AKI.

## Introduction

Acute kidney injury (AKI) is a clinical complication with a high morbidity and mortality rate (1,2). Patients with advanced age, diabetes or vascular diseases are at high risk of AKI. The pathophysiology of AKI is very complex, and includes tubular and vascular cell damage and an intense inflammatory reaction (3). Current therapies for AKI mainly include supportive care and renal replacement therapy. Despite these therapies, the five-year mortality rate for patients with AKI remains >50% (4). Hence, it is important to develop novel therapeutic interventions for improving survival outcomes for patients with AKI.

Stem cell-based therapy has sparked great interest in AKI treatment over the years. A number of studies have demonstrated that stem cells can prevent and repair damage to renal tubular cells in AKI induced by ischemia-reperfusion (IR) (5–7), or chemicals such as cisplatin (8–10) and glycerol (11,12). Different types of stem cells, such as embryonic stem cells (13), amniotic fluid stem cells (14), hematopoietic stem and progenitor cells (15), and mesenchymal stem cells (MSCs) (5,9,10), have been investigated and have shown to be promising in AKI treatment. Among the different types of stem cells, bone marrow-derived MSCs (BM-MSCs) have gained great popularity. BM-MSCs are multipotent cells that can be easily isolated from bone marrow and expanded *ex vivo*(16). As BM-MSCs can be isolated from the bone marrow of patients, their source and safety are well known. Several studies have used BM-MSCs to treat AKI in animal models and have found that renal function and structure can be improved by infusion with BM-MSCs (8,9,17,18). Despite evidence for the therapeutic potential of BM-MSCs, the mechanisms underlying the improvement in kidney function and structure remain unclear.

Previous studies have indicated that apoptotic proximal tubular cell death is a prominent and characteristic feature of AKI induced by cisplatin (19,20). Certain evidence indicates that while cisplatin enhances the number of apoptotic cells in the kidneys, the infusion of MSCs reduces the number of apoptotic cells (9,10,21). Several pathways are involved in cisplatin-induced AKI. Mitogen-activated protein kinase (MAPK) signaling pathways play an important role in cisplatin-induced renal injury. Both p38 and extracellular signal-regulated kinase (ERK) are activated in cisplatin-induced AKI (22,23). Treatment with cisplatin leads to the decrease or degradation of anti-apoptotic proteins, such as Bcl-2, whereas the levels of pro-apoptotic proteins, such as Bax are increased (24).

MSCs have the ability to differentiate into tubular epithelial cells *in vitro*(25). However, integration and differentiation into tubular epithelial cells are rarely reported in experimental AKI models *in vivo*(7). It is generally considered that MSCs promote tubular regeneration mainly through a paracrine mechanism (26). MSCs can secrete several types of growth factors and cytokines, such as vascular endothelial growth factor (VEGF), basic fibroblast growth factor, insulin-like growth factor-1 (IGF-1), interleukin (IL)-6 and IL-11 (27,28). The infusion of MSC-conditioned medium (CM) has been reported to improve renal function and prolong life span in cisplatin-induced AKI (29). For example, heme oxygenase-1 (HO-1)^++^ MSC-CM has been shown to protect against cisplatin-induced renal injury, while HO-1^−/−^ MSC-CM failed to induce renoprotective effects (30). Another study indicated that the knockdown of IGF-1 expression in MSCs by small-interfering RNA (siRNA) led to a significant decrease in the protective effects of MSCs on nephrotoxicity induced by cisplatin (31).

In order for MSCs to be clinically applied in the treatment of AKI more effectively and safely in the future, further understanding of the mechanisms responsible for the renoprotective effects of MSCs is necessary. In the present study, we hypothesized that the infusion of BM-MSCs alleviates damage to renal tubules in cisplatin-induced AKI by inhibiting apoptotic pathways involved in AKI. First, the renoprotective effects of BM-MSCs on cisplatin-induced renal injury were evaluated *in vivo*. Second, tubular cell apoptosis and proliferation after the infusion of BM-MSCs were evaluated. Third, the expression and activation of proteins related to cisplatin-induced renal cell apoptosis were detected, including p38, ERK, caspase-3, Bax and Bcl-2. Finally, the ability of BM-MSC-CM alone to reduce rat kidney cell apoptosis induced by cisplatin was examined *in vitro*.

## Materials and methods

### Ethics

All experiments were performed using Sprague-Dawley (SD) rats and were conducted according to the Wuhan University Guide for the Care and Use of Laboratory Animals. All experimental animal procedures were approved by the Animal Care and Use Committee of Wuhan University, Wuhan, China.

### Preparation of BM-MSCs

MSCs were collected from three- to four-week-old, male SD rats (80–100 g). Briefly, the rats were euthanized by cervical dislocation and their tibias and femurs were cleared of muscle and connective tissue. Bone marrow cells were aspirated using an 18-gauge needle with phosphate-buffered saline (PBS) and passed through a 70-μm nylon gauze (BD Pharmingen, Bedford, MA, USA). The cells were washed twice for 5 min each by centrifugation at 150 × g and resuspended in Dulbecco’s modified Eagle’s medium (DMEM; Gibco, Carlsbad, CA, USA) supplemented with 10% heat-inactivated fetal bovine serum (FBS; Sigma Chemical Co., St. Louis, MO, USA), 2 mM glutamine (Gibco) and penicillin/streptomycin (100 U/ml; Gibco). The bone marrow was plated on 25 cm^2^ culture flasks (Corning; Corning, NY, USA) at a density of 2×10^6^ cells/ml. The flasks were incubated at 37°C with 5% CO_2_ in a humidified atmosphere. Four days after plating, the medium was replaced to remove free-floating cells, and then replenished every two to three days. After growing to 80–90% confluence, the cells were washed with PBS and incubated with trypsin (Gibco) for 5 min at 37°C. Trypsin was neutralized by adding fresh complete medium. The cellular suspension was diluted 1:2 at each passage. DMSO (final concentration, 10%) was added to the cells and they were then frozen in aliquots and stored in liquid nitrogen.

### Phenotypic analysis of BM-MSCs

Flow cytometric analysis was performed to characterize the phenotype of MSCs. Cell suspensions were washed twice with PBS containing 0.1% bovine serum albumin (BSA; Sigma Chemical Co.). For direct assays, aliquots of cells at a concentration of 1×10^6^ cells/ml were incubated at 4°C for 30 min with the following antibodies: fluorescein isothiocyanate (FITC)-conjugated CD34 (Santa Cruz Biotechnology, Santa Cruz, CA, USA), FITC-conjugated CD45 (eBioscience, San Diego, CA, USA), PE-conjugated CD44 (Santa Cruz Biotechnology) and PE-conjugated CD29 (eBioscience). Rat immunoglobulin G1-FITC and rat immunoglobulin G1-PE (BD Biosciences Pharmingen) were used as an isotype-matched control. Labeled cells were analyzed by a FACSCalibur flow cytometer with the use of CellQuest software (Beckman Coulter, Brea, CA, USA).

### Cell lineage differentiation

The adipogenic and osteogenic differentiation potential of the BM-MSCs was investigated at the third passage. To induce adipogenic differentiation, the BM-MSCs were incubated at 100% confluency with rat BM-MSC adipogenic induction medium (Cyagen, Guangzhou, China) for three days and maintenance medium (Cyagen) for one day. After three cycles of induction and maintenance, the cells were cultured in maintenance medium for an additional seven days by replacing the medium every three days. Oil Red O staining (Sigma Chemical Co.) was used to detect lipid vacuoles. For osteogenic differentiation, the BM-MSCs were grown with rat BM-MSC osteogenic differentiation medium (Cyagen) which was replaced every three days. After two to three weeks of differentiation, calcium deposits were stained with Alizarin red (Sigma Chemical Co.).

### Treatment of animals

Male SD rats (weighing, 190–220 g) were used in the experiments. During the experiments, the animals were housed under standard conditions with a 12-h light/12-h dark cycle. Water and food were provided *ad libitum*. AKI was induced in the SD rats by an intraperitoneal injection of 6 mg cisplatin/kg body weight (Sigma Chemical Co.) (32). To investigate the effects of BM-MSCs in the cisplatin-induced AKI animal model, the rats were divided into the following three groups: i) the control group, which received a single intraperitoneal injection of saline (n=8); ii) the cisplatin group, which received a single intraperitoneal injection of cisplatin and was administered 500 μl saline by tail vein injection on day 1 after the cisplatin injection (n=8); and iii) the cisplatin and BM-MSC group, which received a single intraperitoneal injection of cisplatin and was administered BM-MSCs (1×10^6^ cells/500 μl) by tail vein injection 24 h after the cisplatin injection (n=8). All animals were sacrificed on day 4 after the cisplatin injection. Blood, urine and tissue samples were collected for the determination of renal function and tissue damage.

### Determination of renal function

Serum levels of creatinine, blood urea nitrogen (BUN), urine levels of creatinine and urinary microalbumin levels were measured using standard diagnostic kits in an autoanalyzer (Olympus AU400; Olympus, Tokyo, Japan).

### Renal histology

Kidney specimens from all animals were fixed in 10% buffered formalin and embedded in paraffin. Tissues were cut into 5-μm-thick slices and then stained with hematoxylin and eosin (H&E) for light microscopic analysis. Tubular injury was defined as tubular epithelial necrosis, cast formation, tubular dilatation and the loss of the brush border. Tubular injury was scored by grading the percentage of affected tubules under ten randomly selected, non-overlapping fields (magnification, ×200) as follows: 0, 0%; 1, ≤10%; 2, 11–25%; 3, 26–45%; 4, 46–75%; and 5, 76–100%. To score injured tubules, whole tubule numbers per field were considered as standard under a magnification of ×200. The grading percentage was calculated in each field as follows: injury score (%) = (number of injured tubules/number of whole tubules) ×100.

### Apoptosis assay

Apoptosis was determined by a terminal deoxynucleotidyl transferase dUTP nick end-labeling (TUNEL) assay kit (Roche, Indianapolis, IN, USA). Accordingly, the kidney sections were deparaffinized, rehydrated, digested with proteinase K and labeled with a TUNEL reaction mixture for 60 min at 37°C. TUNEL-positive, apoptotic tubular epithelial cells were counted in ten high-power (x400) fields per section in the cortex.

### Proliferation assay

To determine the number of proliferating tubular cells, the expression of proliferating cell nuclear antigen (PCNA) was detected by immunohistochemistry. Paraffin-embedded kidney sections were first deparaffinized with xylene and rehydrated in a series of alcohol and water. After blocking with goat serum for 30 min, the slides were incubated with an anti-PCNA antibody (1:200; Sigma Chemical Co.) at 4°C overnight and anti-rabbit secondary antibody (1:200; Santa Cruz Biotechnology) for 1 h. PCNA signal was detected using a diaminobenzidine (DAB) kit (Beyotime, Haimen, China). Nuclei were visualized by counterstaining with Harris’s hematoxylin. Scoring for PCNA-positive cells was carried out by counting the number of positive nuclei in the renal cortex in ten high-power (x400) fields per section.

### Labeling and preparation of BM-MSCs for transplantation

To examine intrarenal localization and to quantify the BM-MSCs, the BM-MSCs were labeled with a PKH-26 red fluorescence cell linker kit (Invitrogen, Carlsbad, CA, USA) one day after the cisplatin injection and were then infused into the cisplatin-treated rats (n=5). Labeling efficacy was >98%, as assessed by flow cytometry. Viability was >96%, as assessed by trypan blue exclusion. After four days, the rats were sacrificed and samples were immediately frozen in liquid nitrogen, embedded in optimal cutting temperature (OCT) compound, sliced into 5-μm-thick sections, fixed in acetone (10 min) and incubated with FITC-labeled wheat germ agglutinin (WGA Lectin; Vector Laboratories, Burlingame, CA, USA) for 30 min. Nuclei were stained with 4,6-diamidino-2-phenylindole dihydrochloride hydrate (DAPI; Sigma Chemical Co.). PKH-26-positive cells were counted in six frozen renal sections per rat (n=5). Data are expressed as the number of PKH-26-positive cells per renal section.

### Western blot analysis

Immunoblot analysis was performed as previously described (33). Briefly, the tissues or cultured cells were lysed in a buffer containing 20 mM Tris (pH 7.4), 150 mM NaCl, 1 mM EDTA, 1 mM EGTA, 1% Triton X-100, 25 mM sodium pyrophosphate, 1 mM NaF, 1 mM β-glycerophosphate, 0.1 mM sodium orthovanadate, 1 mM phenylmethyl sulfonyl fluoride, 2 μg/ml leupeptin and 10 μg/ml aprotinin. In all, 50 μg of total cell lysate were separated on SDS-PAGE gels and transferred onto polyvinylidene difluoride (PVDF) membranes (Millipore; Billerica, MA, USA). The membranes were blocked with 5% non-fat dry milk in Tris-buffered saline and Tween-20 (TBS-T) buffer and then incubated with the following primary antibodies: phosphorylated ERK (p-ERK) 1:1,000; ERK, 1:1,000; phosphorylated p38 (p-p38), 1:1,000; p38, 1:1,000; Bcl-2, 1:1,000; Bax, 1:1,000; and caspase-3, 1:1,000 (Cell Signaling Technology, Beverly, MA, USA); β-actin, 1:5,000 (Santa Cruz Biotechnology, Santa Cruz, CA, USA) at 4°C overnight. The samples were then incubated for 1 h at room temperature with horseradish peroxidase (HRP)-conjugated anti-rabbit secondary antibody (1:5,000; Santa Cruz Biotechnology) as a secondary antibody. Signals were detected using an ECL Western Blotting Kit (Amersham Pharmacia Biotech, Piscataway, NJ, USA). For quantification, the density of the bands was measured using Quantity One software (Bio-Rad, Hercules, CA, USA).

### Collection of BM-MSC-CM

The BM-MSCs were cultured in a 75-cm^2^ culture flasks in 15 ml complete medium. The medium was changed for 15 ml fresh complete medium when the cells grew to 50% confluence and was collected 72 h later when the cells were 90% confluent. Floating cells were removed by centrifugation at 3,000 rpm for 10 min and the supernatant was frozen at −80°C for use in later experiments.

### Effects of BM-MSC-CM on NRK-52E cell viability and apoptosis

NRK-52E cells (ATCC, Manassas, VA, USA), a rat renal proximal tubular cell line, were cultured in DMEM (Gibco; Carlsbad, CA, USA) supplemented with 10% heat-inactivated fetal bovine serum (FBS; Gibco), 2 mM glutamine (Gibco) and penicillin/streptomycin (100 U/ml; Gibco). When the cultured cells were grown to 80% confluence, the cells were incubated in CM or complete medium in the presence or absence of cisplatin at a final concentration of 50 μM for 24 and 48 h (34). Subsequently, a water-soluble tetrazolium salt-1 (WST-1) assay (29), Annexin V and propidium iodide staining and western blot analysis were performed.

The viability of the NRK-52E cells was assessed using a WST-1 assay kit (Beyotime, Haimen, China). Briefly, the cells were plated in 96-well tissue culture plates. To each well was added 10 μl WST-1 solution at 24 or 48 h after treatment with cisplatin. Following incubation with WST-1 solution for 2 h, the absorbance values were measured at a wavelength of 450 nm.

The percentage of apoptotic cells was assessed by Annexin V and propidium iodide staining (Annexin V-FITC apoptosis detection kit; BestBio, Shanghai, China) by flow cytometry. Briefly, both adherent and floating cells were collected and resuspended in 400 μl of 1X binding buffer. Annexin V-FITC (5 μl) was then added and the cells were incubated in the dark at 4°C for 15 min. Subsequently, 10 μl of propidium iodide were added and the solution was incubated in the dark at 4°C for 5 min. The cells were analyzed by a FACSCalibur flow cytometer (Beckman Coulter) using a 488 nm excitation wavelength for the Annexin V-FITC-positive cells and propidium iodide-positive cells.

### Statistical analysis

Data are expressed as the means ± SEM. Data were analyzed using a t-test and one-way analysis of variance (ANOVA). Statistical analysis was performed using SPSS 13.0 software (IBM; Chicago, IL, USA). A value of P<0.05 was considered to indicate a statistically significant difference.

## Results

### Morphology, immunophenotype and differentiation status of BM-MSCs

**Figure 1:**
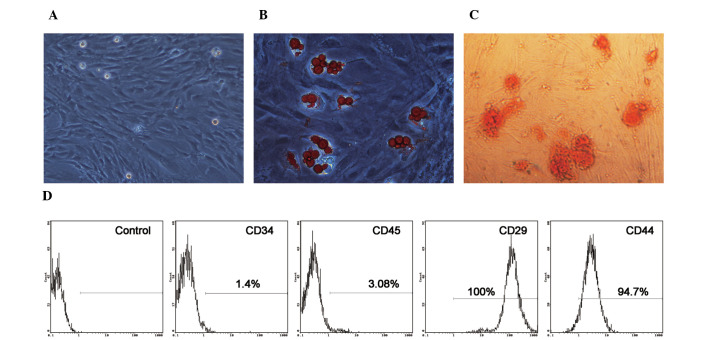
Characterization of rat bone marrow-derived mesenchymal stem cells (BM-MSCs). (A) Representative phase-contrast microscopy image of BM-MSCs with typical spindle-shaped ‘fibroblastoid’ morphology. Original magnification, ×100. (B) Adipogenic differentiation of BM-MSCs stained with Oil Red O. Original magnification, ×400. (C) Osteogenic differentiation of BM-MSCs stained with Alizarin red. Original magnification, ×400. (D) Flow cytometric characterization of BM-MSCs. The hematopoietic cell markers, CD34 and CD45, were weakly expressed. The BM-MSC-specific markers, CD29 and CD44, were strongly expressed on the cells.

The BM-MSCs were isolated and expanded from four-week-old male rats. MSCs cultured as plastic-adherent cells showed a flattened and spindle-shaped morphology *in vitro*. The morphological features of the MSCs are shown in Fig. 1A. To verify the pluripotential capacity of the cultured cells, the cells were exposed to the appropriate induction medium. The BM-MSCs differentiated into adipogenic and osteoblastic lineages two to three weeks after induction (Fig. 1B and C). The expression of cell surface markers on BM-MSCs was evaluated by flow cytometry. As illustrated in Fig. 1D, the BM-MSCs weakly expressed CD45 and CD34, which are two specific cell surface markers of hematopoietic cells. The cells also strongly expressed CD29 and CD44, which are important cell surface markers of MSCs. These results demonstrate the spindle-shaped morphology, purity and differentiation potential of the MSCs (35).

### Infusion of BM-MSCs preserves renal function in rats with cisplatin-induced AKI

**Figure 2:**
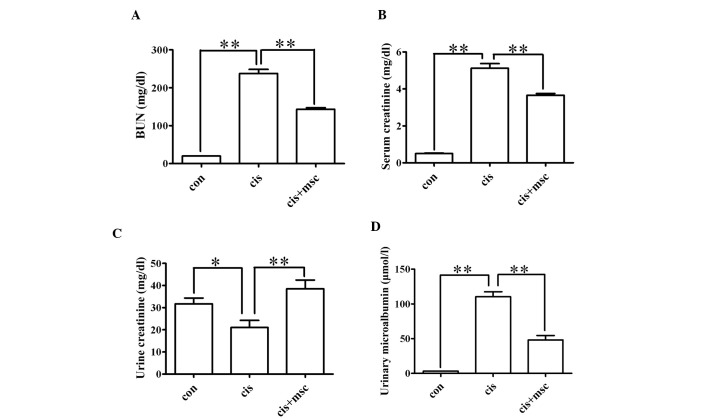
Protective effects of bone marrow-derived mesenchymal stem cells (BM-MSCs) on renal function in rats with acute kidney injury (AKI). Rats were divided into three groups: the control group (con, n=8), cisplatin-treated rats administered saline (cis, n=8) and cisplatin-treated rats administered BM-MSCs (cis + msc, n=8). Renal function was assessed by measuring blood urea nitrogen (BUN), serum creatinine, urinary creatinine and urinary microalbumin levels. Data are presented as the means ± SEM. (A) Serum levels of BUN; con vs. cis, ^**^P<0.01 between the indicated groups; cis vs. cis + msc, ^**^P<0.01 between the indicated groups. (B) Serum levels of creatinine; con vs. cis, ^**^P<0.01 between the indicated groups; cis vs. cis + msc, ^**^P<0.01 between the indicated groups. (C) Urine levels of creatinine; con vs. cis, ^*^P<0.05 between the indicated groups; cis vs. cis + msc, ^**^P<0.01 between the indicated groups. (D) Urine levels of microalbumin; con vs. cis, ^**^P<0.01 between the indicated groups; cis vs. cis + msc, ^**^P<0.01 between the indicated groups.

The cisplatin-treated rats administered saline or BM-MSCs were sacrificed four days after the cisplatin injection. The control rats were also sacrificed four days after the saline injection. Serum and urine samples were collected for the analysis of serum BUN, serum creatinine, urinary creatinine and urinary microalbumin levels. As shown in Fig. 2, the BM-MSCs exerted a renoprotective effect, as reflected by significantly lower BUN, serum creatinine and urinary microalbumin levels (P<0.01) and higher urinary creatinine levels (P<0.05) compared with the cisplatin-treated rats adminstered saline. These data indicate that the infusion of BM-MSCs preserves renal function in rats with cisplatin-induced AKI.

### Infusion of BM-MSCs ameliorates cisplatin-induced renal tubular lesions

**Figure 3:**
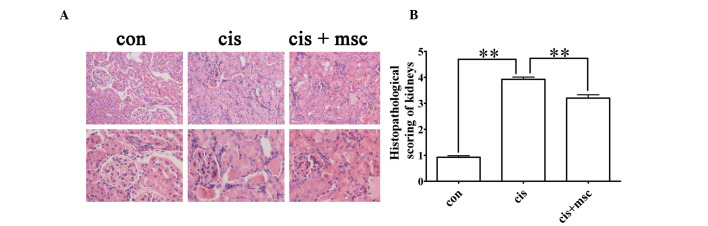
Histological changes and scoring of kidney sections from the control and cisplatin-treated rats administered saline or bone marrow-derived mesenchymal stem cells (BM-MSCs), respectively, four days after the cisplatin injection. (A) Representative hematoxylin and eosin (H&E) staining of kidney sections from the control, cisplatin and cisplatin + BM-MSCs group. Original magnification of images on upper panel, ×200; original magnification of images from lower panel, ×400. (B) Histopathological scoring of cisplatin-induced renal injury. Tubular injury was defined as tubular necrosis, cast formation, loss of brush border in renal tubules and tubular dilatation. Histopathological scoring was based on the percentage of affected tubules in the kidney sections, as described in ‘Materials and methods’. Data are presented as the means ± SEM. con vs. cis, ^**^P<0.01 between the indicated groups; cis vs. cis + msc, ^**^P<0.01 between the indicated groups.

To evaluate the effects of the infusion of BM-MSCs on histological impairment in the kidneys induced by cisplatin, all the rats were sacrificed four days after the cisplatin injection and the kidneys were collected for H&E staining. The kidneys of the cisplatin-treated rats administered saline showed typical cisplatin-induced renal injury, including tubular necrosis, cast formation, the loss of brush border in renal tubules and tubular dilatation (Fig. 3A). The infusion of BM-MSCs significantly attenuated renal tubular damage, as reflected by the light microscopy images and much lower histopathological scoring compared with the cisplatin-treated rats administered saline (P<0.01; Fig. 3A and B). These results suggest that the infusion of BM-MSCs significantly protects the kidneys from cisplatin-induced renal histological damage.

### BM-MSCs reduce apoptosis and accelerate tubular cell regeneration in cisplatin-treated rats

**Figure 4:**
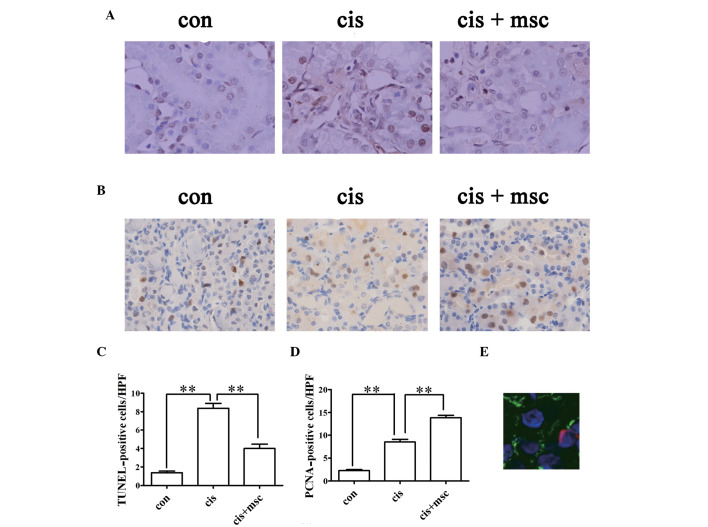
Effects of infusion of bone marrow-derived mesenchymal stem cells (BM-MSCs) on cisplatin-induced tubular apoptosis and tubular cell proliferation four days after the cisplatin injection. (A) Representative images of terminal dexynucleotidyltransferase-mediated dUTP nick end-labeling (TUNEL) staining in the kidney sections from the control and cisplatin-treated rats administered saline or BM-MSCs. Original magnification, ×400. (B) Representative images of proliferating cell nuclear antigen (PCNA) in the kidney sections from the control and cisplatin-treated rats given administered or BM-MSCs. Original magnification, ×400. (C) Quantification of apoptotic cells in kidney sections, identified by TUNEL assay. Data are presented as the means ± SEM. con vs. cis, ^**^P<0.01 between the indicated groups; cis vs. cis + msc, ^**^P<0.01 between the indicated groups. (D) Quantification of PCNA-positive tubular cells in kidney sections. Data are presented as the means ± SEM. Con vs. cis, ^**^P<0.01 between the indicated groups; cis vs. cis + msc, ^**^P<0.01 between the indicated groups. (E) Representative image of PKH-26-labeled MSCs in the kidney sections from rats injected with PKH-26-labeled BM-MSCs four days after the cisplatin injection. PKH-26 labeled cells were stained red. Sections were stained with fluorescein isothiocyanate (FITC)-labeled lectin wheat germ agglutinin (WGA; green), and 4,6-diamidino-2-phenylindole dihydrochloride hydrate (DAPI) for nuclei (blue). Original magnification, ×630.

The apoptosis of renal tubular cells is partly responsible for cisplatin-induced AKI. Using TUNEL assay to detect apoptotic renal tubular cells in the kidney sections, the number of TUNEL-positive cells markedly increased in the cisplatin-treated rats administered saline four days after the cisplatin injection compared with the control rats (P<0.01; Fig. 4A and C). The infusion of BM-MSCs significantly reduced the number of TUNEL-positive cells as compared with the cisplatin-treated rats administered saline on day 4 (P<0.01; Fig. 4A and C).

Tubular cell regeneration was evaluated by PCNA staining. PCNA-positive cells increased in the renal sections from the cisplatin-treated rats administered saline four days after the cisplatin injection compared with the control group (P<0.01; Fig. 4B and D). The number of PCNA-positive cells in the cisplatin-treated rats administered BM-MSCs was much higher than that in the cisplatin-treated rats administered saline four days after the cisplatin injection (P<0.01; Fig. 4B and D). These results indicate that BM-MSCs promote regeneration and exert an anti-apoptotic effect in cisplatin-induced nephrotoxicity.

### Distribution of BM-MSCs infused in cisplatin-treated rats

MSCs have the capacity of migrating into the injured tissue (12). The existence of BM-MSCs in the renal parenchyma was evaluated by the presence of PKH-26-labeled cells in the kidney sections three days after the administration of BM-MSCs. PKH-26-positive cells were counted and were observed in 3±0.5 out of 10^5^ renal cells in renal tissues of cisplatin-treated rats on day 4. MSCs were predominantly localized in the peritubular areas and rarely within the tubular epithelium (Fig. 4E), which suggested that it is unlikely that the MSCs differentiated into renal cells.

### Infusion of BM-MSCs decreases the phosphorylation of MAPKs, including p-ERK and p-p38 in the cisplatin-treated rats

**Figure 5:**
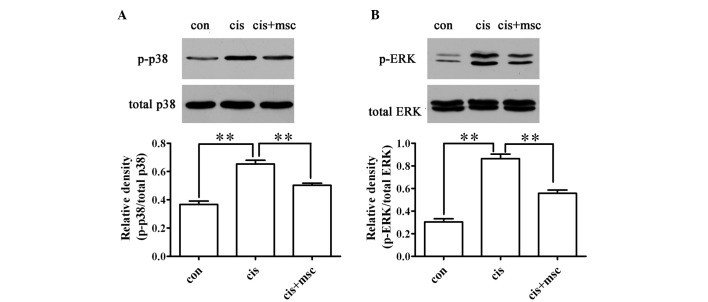
Effects of bone marrow-derived mesenchymal stem cells (BM-MSCs) on the activation of extracellular signal-regulated kinase (ERK) and p38 four days after the cisplatin injection. (A) Expression of phosphorylated p38 (p-p38) detected by immunoblot analysis (n=3 per lane). Total p38 (t-p38) was used as a loading control. The relative density of p-38 to t-38 was compared. Data are presented as the means ± SEM, ^**^P<0.01. (B) Expression of phosphorylated ERK (p-ERK) detected by immunoblot analysis (n=3 per lane). Total ERK (t-ERK) was used as a loading control. The relative density of p-ERK to t-ERK was compared. Data are presented as the means ± SEM, ^**^P<0.01.

The phosphorylation of ERK and p38 has been demonstrated to be involved in cisplatin-induced renal tubular cell apoptosis (22). As shown in Fig. 5, the infusion of BM-MSCs inhibited the phosphorylation of ERK and p38 compared with the cisplatin-treated rats administered saline. These results suggest that BM-MSCs reduce renal tubular apoptosis by inhibiting the activation of ERK and p38.

### BM-MSCs influence the expression of apoptosis-related proteins, such as Bax and Bcl-2

**Figure 6:**
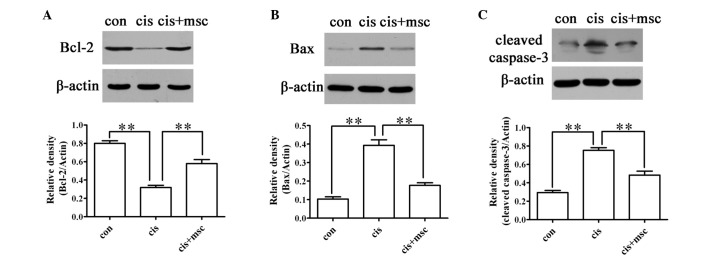
Expression levels of apoptosis-related proteins measured by western blot analysis four days after the cisplatin injection. (A) Expression of Bcl-2 with β-actin as a loading control (n=3 per lane). The relative density of Bcl-2/actin was compared. Data are presented as the means ± SEM, ^**^P<0.01. (B) Expression of Bax with β-actin as a loading control (n=3 per lane). The relative density of Bax to β-actin was compared. Data are the means ± SEM, ^**^P<0.01. (C) Expression of cleaved caspase-3 with β-actin as a loading control (n=3 per lane). The relative density of cleaved caspase-3 to β-actin was compared. Data are presented as the means ± SEM, ^**^P<0.01.

The pro-apoptotic gene, Bax, and the anti-apoptotic gene, Bcl-2, play key roles in cisplatin-induced AKI (36). Caspase-3 acts as an executioner protein in the apoptotic pathway. The downregulation of Bax and caspase-3 and the upregulation of Bcl-2 were observed in the cisplatin-treated rats administered BM-MSCs as compared with the cisplatin-treated rats administered saline (Fig. 6). These results demonstrate that another mechanism through which BM-MSCs reduce cell apoptosis in cisplatin-treated rats is by regulating the expression of Bax and Bcl-2.

### Effects of BM-MSC-CM on NRK-52E cell viability and apoptosis in vitro

**Figure 7:**
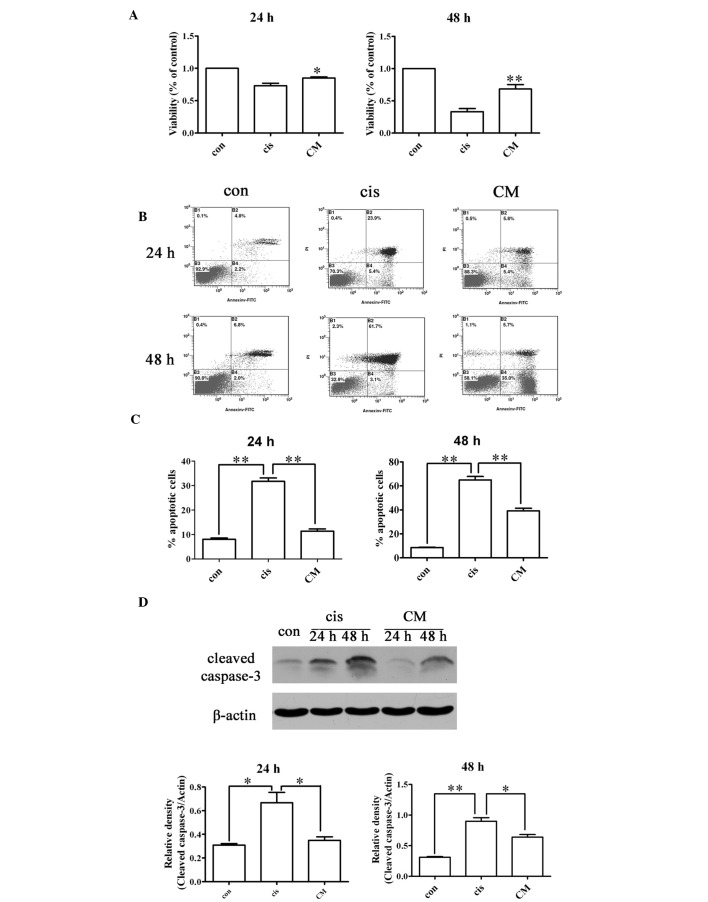
Protective effects of conditioned medium (CM) derived from bone marrow-derived mesenchymal stem cells (BM-MSCs) on NRK-52E cells. NRK-52E cells were exposed to 50 μM cisplatin with BM-MSC-CM or complete medium (cis) for 24 and 48 h. (A) Viability of NRK-52E cells assessed by WST-1 assay 24 and 48 h following treatment with cisplatin. Data are presented as the means ± SEM, ^*^P<0.05, ^**^P<0.01. (B) Representative images of FACS analysis staining with Annexin V and propidium iodide in normal cells (con, n=3), cisplatin-treated cells with (cis, n=3) and without BM-MSC-CM (n=3). (C) Percentage of apoptotic cells assessed by Annexin V and propidium iodide staining. Data are presented as the means ± SEM, ^**^P<0.01. (D) Expression of cleaved caspase-3 with β-actin as a loading control measured by western blot analysis (n=3 per lane). The relative density of cleaved caspase-3 to β-actin was compared. Data are presented as the means ± SEM, ^*^P<0.05, ^**^P<0.01.

To investigate the effects of BM-MSC-CM on cisplatin-treated NRK-52E cells, the NRK-52E cells were exposed to 50 μM cisplatin in the presence or absence of BM-MSC-CM for 24 or 48 h. The viability of the cisplatin-treated NRK-52E cells exposed to CM was significantly higher than those not exposed to CM (P<0.01; Fig. 7A).

As illustrated in Fig. 7B and C, BM-MSC-CM reduced the percentage of apoptotic cells compared with the cisplatin-treated NRK-52E cells in the absence of CM (P<0.01), as shown by flow cytometry analysis (FACS) following Annexin V and propidium iodide staining. BM-MSC-CM also reduced the expression of cleaved caspase-3 in the cisplatin-treated NRK-52E cells (Fig. 7D). The addition of BM-MSC-CM alone was able to reduce renal tubular cell apoptosis. These data indicate that BM-MSCs secrete factors that provide renal protection, thereby attenuating cisplatin-induced renal nephrotoxicity by inducing paracrine effects *in vivo*.

## Discussion

BM-MSCs are multipotent stem cells with immunomodulatory ability, the capacity for expanding easily *in vitro* and the potential for differentiation into cells of mesenchymal or other lineage. Such unique properties have made BM-MSCs an attractive candidate for stem cell-based therapy. Previous studies have shown that the infusion of BM-MSCs is effective in treating myocardial infarction (37,38), neurological diseases (39,40), diabetic nephropathy (41,42) and AKI. In agreement with previous studies investigating the effects of BM-MSCs on AKI, this study suggested that the infusion of BM-MSCs improves kidney function and ameliorated renal injury induced by cisplatin. However, little is known at present about the mechanisms underlying the therapeutic effects of BM-MSCs on cisplatin nephropathy. The present study focuses on the anti-apoptotic mechanisms responsible for the therapeutic effects of BM-MSCs in cisplatin nephropathy.

Tubular cell apoptosis is a characteristic feature of cisplatin nephrotoxicity, which results in the loss of renal endothelial cells and renal dysfunction. The apoptosis of renal cells induced by cisplatin has been detected both *in vivo* and *in vitro*(43–45). Several therapeutic interventions targeting apoptotic pathways involved in AKI have demonstrated beneficial effects on renal injury induced by cisplatin in animal models and cultured renal tubular cells (46–48). In the present study, the number of TUNEL-positive cells increased significantly after the cisplatin injection. The infusion of BM-MSCs resulted in a significant reduction of cells positive for TUNEL staining. The decreased cleaved caspase-3 expression also confirmed that the infusion of BM-MSCs reduced apoptosis in the kidneys injured by cisplatin. PCNA expression is an index of renal regeneration. The administration of BM-MSCs significantly increased the number of PCNA-positive cells, suggesting that BM-MSCs strongly promoted tubular cell proliferation while inhibiting cell apoptosis. These findings suggest that the renoprotective effects of BM-MSCs may rely on their anti-apoptotic function.

To further elucidate the anti-apoptotic mechanisms through which BM-MSCs exert renoprotective effects, we examined certain pathways involved in renal cell apoptosis induced by cisplatin. ERK and p38 are members of the MAPK family. The activation of MAPKs regulates cellular homeostasis and processes, such as proliferation, differentiation and apoptosis (49,50). The phosphorylation of ERK and p38 increases in kidney tissue or in cultured renal tubular cells following the administration of cisplatin, while MEK inhibitors or p38 inhibitors attenuate cisplatin-induced renal injury by decreasing apoptosis (22,23,51,52). Our study confirmed the results of previous studies, namely that the phosphorylation of ERK and p38 increased following treatment with cisplatin. The infusion of BM-MSCs resulted in a reduction of ERK phosphorylation and p38 phosphorylation in parallel with a reduction of apoptosis and an improvement of renal function.

Cisplatin also induces outer mitochondrial membrane injury, suggesting that the activation of the mitochondrial pathway of apoptosis plays an important role in cisplatin-induced nephrotoxicity. *In vivo*, the ratio between Bcl-2 and Bax is reduced following treatment with cisplatin. Bax^−/−^ mice are protected from cisplatin-induced renal injury (36,48,53). *In vitro*, treatment with cisplatin leads to a decrease or the degradation of anti-apoptotic proteins, whereas the levels of pro-apoptotic proteins are increased (24). Our study demonstrated that the administration of BM-MSCs upregulated the anti-apoptotic protein, Bcl-2, and downregulated the pro-apoptotic protein, Bax, which corresponded with reduced apoptosis and improved renal function. These results suggest a correlation between the changes in the mitochondrial pathway of apoptosis and the infusion of BM-MSCs.

There are two potential, distinct mechanisms responsible for the renoprotective effects of BM-MSCs against cisplatin-induced nephrotoxicity: transdifferentiation or through a paracrine process. Certain studies have reported that exogenous BM-MSCs can engraft into injured tubules and it has been proposed that the ability of the BM-MSCs to transdifferentiate explains their protective effects (8,11,54). By contrast, other studies have shown that BM-MSCs protect against acute tubular injury through a differentiation-independent process (7,26,55,56). In our study, we observed the renoprotective effects of BM-MSCs only three days after their administration. This is too short a time period for the BM-MSCs to transdifferentiate into renal tubular cells. The distribution of BM-MSCs in kidney sections further confirmed that they had little opportunity to differentiate into renal cells. The injected cells were mainly located in the peritubular areas, not in the content of the tubules. We then examined whether CM derived from BM-MSCs protects renal tubular cells and attenuates cell apoptosis *in vitro*. NRK-52E cells, an immortalized cell line derived from rat proximal tubules, were incubated with cisplatin in the presence or absence of CM. The results revealed that CM alone increased cell viability and reduced cell apoptosis, as assessed by Annexin V and propidium iodide staining and the reduced expression of cleaved caspase-3. These results support the idea that BM-MSCs protect tubular cells by inhibiting cell apoptosis through a paracrine mechanism.

In conclusion, we demonstrate that the infusion of BM-MSCs partially protects cisplatin-treated rats from AKI by inhibiting tubular cell apoptosis. ERK, p38, Bcl-2 and Bax are involved in the anti-apoptotic effects of BM-MSCs in cisplatin nephrotoxicity. Furthermore, BM-MSCs may act by secreting factors (i.e., a paracrine mechanism) that subsequently cause the changes in the expression or activation of key proteins that inhibit apoptosis and promote proliferation. Our study provides a preliminary understanding of the role of BM-MSCs in the treatment of AKI.
